# Introduction to the Study and Practice of Midwifery, and the Diseases of Women and Children

**Published:** 1834-01-01

**Authors:** 


*Introduction to the Study and Practice of Midwifery, and the Diseases of Women and Children.* By WILLIAM CAMPBELL, M.D.—Edinburgh, 1833. 8vo. pp. 714; and 3 plates.

FROM the earliest ages to the present time the diseases of women have been discussed with a minuteness, and commented on with a felicity, which it would be in vain to seek in any other department of medicine. This may be attributed in part to the interest naturally excited by the sufferings of the more agreeable half of the human species, and in part to the reflex operation on the medical profession of the same sentiment existing in the breasts of the public; for, from Chamberlayne to Gooch, the golden showers of public patronage have ever descended in the freest streams on the professors of midwifery. The encouragement given to this branch of physic is, of course, extended by the profession to the works which elucidate it; and hence the enormous number of Systems, Manuals, and Elements of Midwifery, which, to critics less good-humoured than ourselves, might sometimes seem to exceed all reasonable bounds. Yet still it cannot be expected that any one treatise should comprehend all that the student desires to know; and we therefore commend him who, while turning over Denman, "nocturnâ et diurnâ manu," does not shut his eyes to the light which more recent authors have thrown upon the darker passages of this high-priest of Lucina. It is almost unnecessary to observe, that Denman's excellent work is deficient in the physiology of generation, in the etiology and pathology of some important puerperal diseases, the diseases of children, and the peculiarities of infant life. Admirable as the works of Burns and Hamilton confessedly are, they leave much undone, which is the more to be deplored, seeing that what they have achieved is done surpassingly well. Where is the work on uterine hemorrhage comparable to Rigby's? But, half a century has elapsed without any notable addition to his instructions! Without Hey, Armstrong, Gooch, and Marshall Hall, we should be still—as before their valuable contributions—in the Slough of Despond whenever cursed with puerperal epidemics. An essay might be made on the subject, but enough has been said to convince the modest inquirer that his search will be sleeveless, for an entire and perfect chrysolite, in the shape of a system of obstetric medicine. It must not be thought we are ignorant of, or insensible to, the value of the labours of foreign practitioners, but it cannot be contended that they have supplied the defect.

The deficiency has been so long known to all—teachers especially,—that we are unable to say anything excusatory for Dr. Campbell's failing to supply it. Misconceive us not: there is no desire on our part to depreciate his merit, but to ourselves and readers we owe it to declare that many points of great interest are superficially, and not always correctly, dealt with. If his book were as perfect as his index is copious, there would be nothing left to desire. To justify this assertion, prior to a more detailed analysis, let us take a case. The pathology, symptoms, and treatment of "Suppression of

Urine, Retention of Urine, Incontinence of Urine, Diabetes, Swelling of the Breasts, Leucorrhœa, Phimosis, Paraphimosis, Hydrocele, Rachitis, Scrofula, and Continued Fever," are all disposed of in eleven pages! Brevity may be the soul of wit, but surely not also of physic. Observe, that he is thus laconic when treating of the most neglected and least understood of subjects,—the diseases of children; a neglect principally of those who undertake the self-imposed and responsible duty of instructing their successors.

The author's account of conception, generation, and the gravid uterus, is vague and unsatisfactory: he too seldom refers to, and still less often avails himself of, the works of those French and German physiologists to whose researches we owe much previous, and nearly all subsequent illumination, from the days of Hunter.

Recent as is Dr. Campbell's system, he repeats the exploded error that the *decidua* is a membrane peculiar to the uterus, instead of being proper to the ovum. The engraving of the fœtal circulation is very inaccurate. The left kidney is indicated as the right one. The arch of the aorta is apparently omitted, and for it we have what is pointed out as the ductus arteriosus. He insists that the areola of the nipple is an infallible sign of pregnancy: perhaps no case was ever known where it was absent;—it is universal; but there are disturbances of the uterine system simulating some of the characters of pregnancy, and we and others have seen the areola without gravidity. He states, from a limited experience, that the development of the uterus from distention by hydatids is inconsiderable; but cases are recorded, and we have seen one, in which the volume of hydatids and fluid amounted to three gallons.

In Dr. Campbell's opinion of the secale cornutum we concur, aye, even go further. The apocryphal virtues of this medicine have been advocated with a zeal almost amounting to bigotry; and, if the friends of the drug had been inquisitors, some of the doubters would have been persecuted. In the favourable testimony there is no want of integrity, but a sad lack of philosophy; and the *number* of converts is not even a presumptive evidence to us, who are not of the faithful.

The instructions for using the forceps are loose and incomplete, especially the long forceps. These instruments, in their ordinary form, are always difficult of application, and often unsafe to both mother and child; yet he directs, in one position, "the blade of the forceps to be inserted between the pubes and the head;" a proceeding that no testimony could convince us is practicable in the supposed case, *i. e.* when the fœtal head is above the brim of the pelvis, but which, if practicable, would be perilous in the highest degree. Dr. Campbell does not mention the long forceps with blades of unequal length, which modification obviates some of the difficulty of their application, and the danger of their use; nor does he appear to recognize the advantage often gained by having the short forceps with a short blade for occasional use. Substituting a shorter for one of the ordinary blades of this instrument, is frequently of the greatest advantage. It has sometimes happened that a case not manageable by the vectis was denied the benefit of the forceps, because the second blade could not be applied. This difficulty suggested the shorter blade, which gave all the assistance sought, and with increased facility of introduction. We have seen cases thus rescued from the perforator, and in practice have ourselves experienced the advantage of this important improvement.

Although the treatment directed for flooding in the advanced months of pregnancy is generally judicious, he takes some unfounded exceptions to a measure always useful, and sometimes indispensable, namely, plugging the vagina; alleging as his objection that the *internal* hemorrhage may destroy the patient. This is so remotely possible as not to deserve being made an element in the calculation; but, if such apprehension existed, the evil might be averted by external pressure.

There is great want of energy in his instructions for arresting hemorrhage after delivery; and we think the delay he recommends in extracting the placenta, would, if often permitted, lead to disastrous results.

Dr. Campbell enjoys an enviable satisfaction in his own knowledge of puerperal fever, and the success of his treatment; but we are sorry to see him indulge in some less than kind allusions to Dr. Abercrombie, a man who may indeed hold cheap any animadversions of Dr. C. He does not even acknowledge, and perhaps knows not, the value of Dr. Gooch's excellent assistance in the pathology and treatment of the disease. Dr. Armstrong's name is only mentioned, and that for the opportunity it affords of saying something uncivil of Dr. Abercrombie. Mr. Hey is alike neglected. Is this worthy a philosopher? The whole section on puerperal fever is rambling, desultory, and unsatisfying.

Scarcely can we say more for that on puerperal mania, on which subject he forgets to quote Dr. Gooch, who might fairly claim the paternity of the best portion of the section.

"In regard to the proximate cause, we can advance little beyond conjecture. It is a very general opinion that the brain is the seat of disease when it appears under the sthenic form, but no dependence can be placed on any appearance exhibited by this organ, nor on the accounts afforded us by some of the most celebrated anatomists; since, on the one hand, maniacal patients have been known to expire under the most violent disturbance, apparently of the brain, without the slightest lesion of this organ being discovered on dissection; and since, on the other, the cerebral system has been seen universally diseased without any mental derangement. We may take an illustration from the other sex, and instance the extraordinary case of Mr. Kay, No. 226. Lond. Phil. Transac., in whose cheek a cancerous ulcer commenced, destroyed his eye, penetrated the os frontis and dura mater, and continued so long, that gradually the whole brain was consumed, and when he died there was nothing found in the cranium but black putrid matter; yet he lost no sense, nor the motion of any organ, nor had he any convulsion or spasm. In other instances the cranium, on examination, has been found almost completely deprived of its contents; and in some animals it has been filled with ossific matter; yet the functions thought to depend on its integrity were not impaired. From the occurrence of such cases, there is some excuse for those who have assigned the seat of the soul to the stomach, plexus solaris, and other organs; but we may take it for granted, however, that the brain and its emanations are the parts most intimately connected with the intellect, and that some morbid change of these exist in every instance of insanity, though generally so attenuate as to elude our search, in parts whose organization is so delicate and complex. But the brain and nerves are not the only organs, since many others have been found to participate, as the liver frequently in the male, and the uterus in the female sex." (P. 376.)

Can Dr. Campbell seriously believe in the correctness of Mr. Kay's case?

We regret exceedingly that, for pupils, the information contained in this work is inadequate; and it could only be useful to a practitioner of very moderate pretensions. There are numerous examples of eccentric orthography: for some of them perhaps the printer may be responsible, but who will stand sponsor for "mezentary?" Its Greek derivation does not sanction this.

The following is a good specimen of one of Dr. Campbell's happier passages:

"When the child escapes through the oreach in utero, into the abdomen, three modes of practice are recommended; first, to draw the fœtus back into the uterus, and thereafter extract it per vaginam; secondly, to leave matters to nature; and thirdly, to accomplish the delivery by gastrotomy. In regard to the first method, its success has been such, that the practice should not now be sanctioned, since every patient in whom it has been resorted to, whether late or early, fell a victim to it. We need not be much surprised at this, since the uterus, very shortly after it has been injured, must contract to such an extent as to preclude the possibility of its receiving the hand without the employment of force, which will assuredly be followed by an extension of the rent. Doubtless there are examples related, where we are informed the fœtus has been brought back into the uterus, and thence extracted per vaginam, after having been many hours, or several days even, lodged among the intestines; but the author is disposed to believe that, in these instances, the injury was not in the body, but in the cervix uteri, and upper part of the vagina, points which are far less contractile, and of which a laceration is not by any means so formidable, as of the upper parts of the organ.

"In support of the second method, cases have been published where the child, after its escape from the uterus into the abdominal cavity, was permitted to remain therein for years without inconvenience to the parent, who, at some ulterior period, got rid of it by the suppurative process, or who indeed conceived again during its retention, and who, after all, had a complete recovery. Admitting that some individuals have recovered under such extraordinary circumstances, what does this prove? merely that nature is occasionally capable of making efforts for the support of our frame, which could never have been contemplated; and that although, in some rare instances, women have survived such complicated tortures, yet, that we are not justified in trusting such cases to the powers of the system, since it is well known that a large majority of them have sunk under the most painful and protracted sufferings. As to those examples in which we are informed that the sex conceived during the retention of the extra-uterine fœtus, it must be acknowledged that they are rather too marvellous for belief.

"The third method, though very formidable, from its near resemblance to the Cæsarean section, would seem, however, to have been attended with most success. In the Journal de Medicine, vol. iii. for 1768, the first well authenticated case of recovery will be found. The child was still-born. A second successful instance, in which a woman had been twice operated on, is detailed in the Pathol. Chirurg. vol. ii. The second time this expedient was resorted to, the child continued to live half an hour after its extraction. A third successful operation of this nature is related in the Quarterly Journal of Foreign Medicine, vol. ii. The patient had not been operated on for twelve hours after the occurrence of the accident. The fourth successful instance, in which both the mother and child were saved, is detailed in the Edin. Jour. Med. Sci. vol. i. From these cases, the author considers himself, on every occasion in which the extraction of the fœtus has not been attempted almost immediately after the accident, to recommend the section of the abdominal parietes, in preference to either of the other methods. The same practice should also be pursued when the womb has been injured, and the fœtus has escaped among the intestines, before the uterine aperture has either become dilatable, or has been expanded to a sufficient extent to receive the hand, with a view to turning. Where an effusion of liquor amnii or of blood has taken place into the peritoneal sac, this is a further inducement for performing gastrotomy. Lastly, where, after the removal of the fœtus by the natural passage, a portion of intestine protrudes the breach in the uterus, and where this organ has contracted to such extent that the hand could not be received to reduce the protruded viscus, Pigrai, the friend and pupil of Ambrose Paré, recommended the section of the abdominal parietes, which, though the event in the case recorded by Dr. M'Keever was successful, seems unavoidable.

"Finally, as in some females, who survived rupture of the uterus, the same accident has been known to happen to them in their succeeding labours, it has been proposed, in 1709, by Dr. Douglas, of London, as stated in a former place, to extract the fœtus by the feet, in women who had once been thus unfortunate; and the proposition is one which has frequently since been acted on with success." (P. 318.)

Dr. Campbell's estimate of the nitrate of silver injection is neither candid nor correct. "Of late years much has been said of the nitrate of silver in solution for the cure of leucorrhœa, but it does not appear to be possessed of greater virtues than many other remedies better known to the profession."

We now come to a graver matter. Treating of Hydrocephalus Acutus, he says, "Of late years, Professor Monro, in the university of Edinburgh, has, with his wonted talent, written a work on this subject, wherein he contends for debility as the principal cause; *but the very rare occurrence of effusion into the brain, after extreme prostration in consequence of disease, is a sufficient refutation of this doctrine.*" Is the doctor dreaming? Can he be ignorant of the fact with which the cultivators of morbid anatomy are so familiar, that in children chronic disease rarely, if ever, terminates fatally without first producing effusion within the cavities of the brain? How much do we owe to Drs. Marshall Hall, and Gooch, for insisting so strongly on the fact, that nothing tends more to cerebral effusion than debilitating disease, or excessive depletion! Can Dr. Campbell be aware of these circumstances, now established to demonstration?

For the same purpose that beacons are raised do we quote the following passage, premising that it contains advice so mischievous, that we cannot persuade ourselves the author was serious when he wrote it: "But it would seem most prudent in every case that exhibits great cerebral excitement, to treat it as one of inflammation of the brain, since, though not actually so at the onset, it might, by inefficient treatment, ultimately terminate in this way." Our only commentary shall be, to ask Dr. Campbell if he doubts that an accelerated circulation through an exhausted sensorium will produce "cerebral excitement?" and whether, as this is the most common condition in the latter stages of adynamic diseases, he would treat them as cases of inflammation of the brain?

